# Real-Life Management of Patients with Retinal Vein Occlusion Using I-Macula Web Platform

**DOI:** 10.1155/2017/5601786

**Published:** 2017-07-24

**Authors:** Massimo Nicolò, Monica Bonetto, Raffaella Rosa, Donatella Musetti, Maria Musolino, Carlo Enrico Traverso, Mauro Giacomini

**Affiliations:** ^1^Clinica Oculistica-DINOGMI, Università di Genova and Italy Ospedale Policlinico San Martino, Genoa, Italy; ^2^Fondazione per la Macula Onlus-Genova, Genoa, Italy; ^3^Healthropy SRL, Genoa, Italy; ^4^DIBRIS, University of Genoa, Genoa, Italy

## Abstract

**Aim:**

*Real-life* evaluation in the management of patients affected by macular edema secondary to retinal vein occlusion.

**Material and Methods:**

A retrospective, observational study using the *I-Macula Web* platform.

**Results:**

Thirty-five patients (37 eyes; 15 females and 20 male) affected by RVO were analysed. At 12 months, there was a statistically significant improvement of best-corrected visual acuity (*p* = 0.0235) and central macular thickness (*p* < 0.0001). The mean change in visual acuity was 8.9 letters. Twenty-seven eyes underwent DEX implant (*n* = 62; mean: 2.29) only. Of these, 8, 4, 14, and 1 eyes underwent 1, 2, 3, and 4 DEX implants, respectively. The remaining 10 eyes were also injected with ranibizumab (*n* = 49; mean: 4.9). At 12 months, 12 eyes (32.5%) presented a dry macula, whereas the remaining 25 eyes (67.5%) still had macular edema. Mean interval between the first and second treatment (T1) and between the second and third treatment (T2) were 5.15 and (T2) 3.7 months, respectively. Where only DEX implants were received, T1 and T2 was 5.1 and 4.9 months, respectively.

**Conclusions:**

This study confirms that DEX implants and/or anti-VEGF drugs improve visual acuity and central macular thickness in patients affected by RVO.

## 1. Introduction

In industrialised countries, retinal vein occlusion (RVO) is the second most common vascular pathology after diabetic retinopathy [1]. RVO, both central (CRVO) and branch (BRVO), affects the retina and causes loss of visual acuity. In both CRVO and BRVO, macular edema is a major contributor to loss of visual acuity. The onset of retinal ischemia and iris neovascularization may, in time, lead to neovascular glaucoma, especially where CRVO is the case [1]. Although the pathogenesis of macular edema secondary to RVO is, as yet, unclear, the production of anti-inflammatory cytokines (prostaglandins and interleukin-6) and angiogenic factors as vascular endothelial growth (VEGF) and the upregulation of tight junctional proteins resulting from the hydrostatic effects of venous hypertension appear to be the keys [2, 3]. Major risk factors in RVO are systemic arterial hypertension, hypercholesterolemia, diabetes mellitus, and glaucoma [4].

Two classes of currently marketed drugs cater for the treatment of post-RVO macular edema, that is, biodegradable and slow-release implant of dexamethasone (Ozurdex, Allergan) and anti-VEGF drugs ranibizumab (Lucentis, Novartis) and aflibercept (Eylea, Bayer) [5–10].

The study aims to explore real-life and day-to-day management of patients with macular edema secondary to RVO treated with intravitreal treatment with dexamethasone (DEX) implant and/or anti-VEGF drugs in a real-life setting. Clinical data were stored electronically on the *I-Macula Web* platform, specifically designed for the management of patients with degenerative and vascular retinal diseases [11].

## 2. Material and Methods

This retrospective, observational study was conducted at the Medical Retinal Center of the University Eye Clinic of Genova, Italy.

The following inclusion criteria were entered onto the *I-Macula Web* platform:

- Diagnoses of macular edema secondary to RVO
- Injection treatment with DEX implant and/or anti-VEGF drugs
- Last visit after at least a 12-month period of treatment.

Standard procedures at this centre require that patients diagnosed with RVO undergo the following tests: standardized best-corrected visual acuity (BCVA) performed with Early Treatment for Diabetic Retinopathy Study (ETDRS) charts, slit lamp biomicroscopy, tonometry, ophthalmoscopy, SD-OCT, and fluorescein angiography. BCVA, intraocular pressure, biomicroscopy, and SD-OCT were repeated at follow-up visits. Retreatment criteria were based basically on the presence of macular edema. In order to have a real-life frame of the management of RVO patients, the following variables were extracted by the web platform:

- Mean age ± standard deviation (SD), gender, and follow-up (±SD);
- Mean change in number of letters, intended as the mean difference between the number of letters read at baseline and at final follow-up;
- Mean change in central macular thickness (CMT), intended as the mean difference between CMT at baseline and at the final follow-up;
- Percentage of the eyes with an improved visual acuity of ≥15 letters at the final follow-up;
- Percentage of the eyes with loss of visual acuity of ≤15 letters at the final follow-up;
- Exposure to treatment regimen; mean number of injections distinguished by drug type;
- Mean time between diagnosis and the first treatment;
- Average time between the first and the second and between the second and the third intravitreal injection of DEX;
- Percentage of patients presenting macular edema at the last follow-up visit;
- The correlation between macular edema at each follow-up and the undertaken clinical decision.

### 2.1. Injective Treatment

Injective procedures were performed in the operating room after topical anaesthesia with benoxinate hydrochloride eye drops. The DEX implant Ozurdex® (Allergan) and the anti-VEGF ranibizumab Lucentis® (Novartis) drugs were used for this study. Injections were performed 3.5–4 mms from the limbus into the lower temporal sector.

## 3. Results

Thirty-five patients (15 females and 20 males) of 37 eyes with macular edema secondary to RVO were identified and analysed.  Table 1 reports the demography of the selected study population. Mean follow-up time was 13.3 months and mean age was 72 years.  Table 2 reports age-related distribution across the sample. In 11.4% (*n* = 4) of the study population, ages ranged between 40 and 59 years; the remaining 88.6% (*n* = 31) was over 60 years.

Seventeen eyes presented CRVO and 20 presented BRVO. Bilateral RVO occurred in two subjects. In both cases, both the eyes were included in the study. Of the remaining samples, 5 subjects presented other macular diseases in the contralateral eye, and specifically, epiretinal membrane, age-related macular degeneration, and pathological myopia in 2, 2, and 1 eyes, respectively.

### 3.1. Visual Acuity

**Figure 1:**
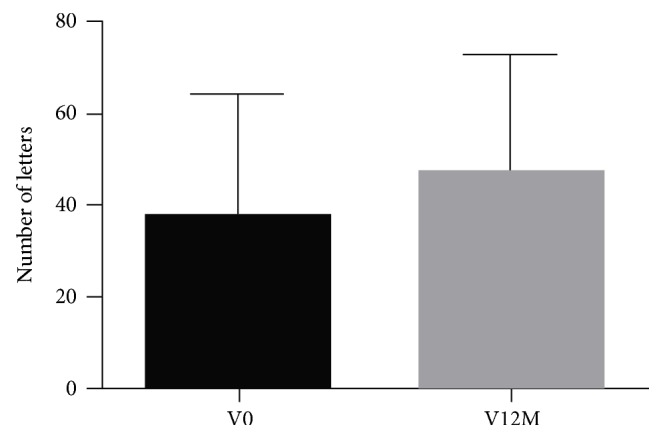
Number of letters at baseline and 12 months in 37 eyes with macular edema secondary to retinal vein occlusion treated with DEX implant and/or ranibizumab.

BCVA in terms of letters read was 37.9 and 46.9 at baseline and at 12 months, respectively (*p* = 0.0235; paired *t*-test) (Table 3 and Figure 1). The mean change at 12 months was 8.9 letters. On the whole, 11 eyes (29.7%) improved more than 15 letters, while 5 eyes (13.5%) worsened by at least 15 letters at 12 months. Within the CRVO or BRVO cohorts, improvement in visual acuity was not statistically significant (Table 2), although the mean change in the number of letters was 8.3 and 9.5 in CRVO and BRVO, respectively.

### 3.2. Central Macular Thickness (CMT)

**Figure 2:**
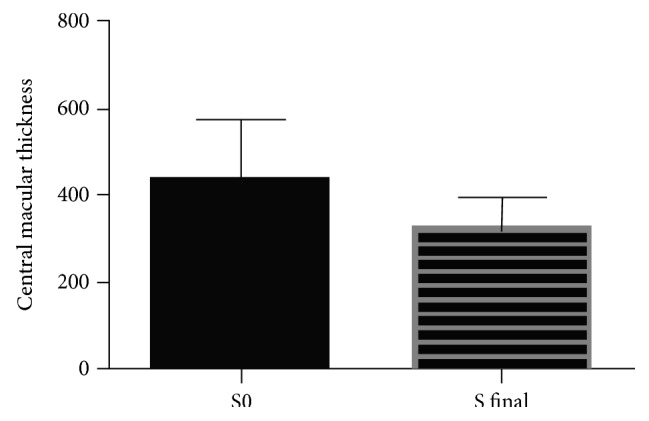
Central macular thickness at baseline and 12 months in 37 eyes with macular edema secondary to retinal vein occlusion treated with DEX implant and/or ranibizumab.

Mean CMT was 436.3 and 322.2 microns at baseline and at 12 months, respectively (*p* < 0.0001; paired *t*-test) and the mean change of CMT at 12 months was −22.51 microns. Within the CVRO and BRVO cohorts, CMT decrease was statistically significant with a mean difference of −151.6 and −87.86 microns, respectively (Table 4 and Figure 2).

### 3.3. Exposure to Drugs and Administration Regimen

**Figure 3:**
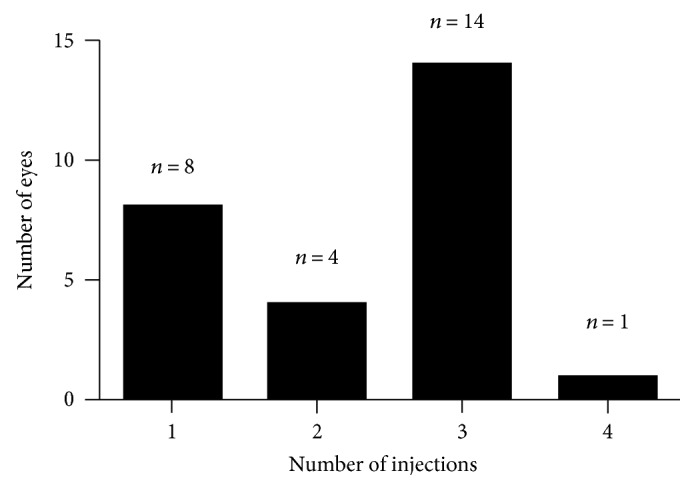
Number of injections in 27 eyes who received DEX implant.

Throughout the follow-up, a total of 111 intravitreal injections were administered for a mean of 2.36 injections per eye (Table 5). DEX-based first-line therapy was selected for all the eyes. Of the 27 eyes injected solely with DEX implant (*n* = 62; mean: 2.29), 8, 4, 14, and 1 eyes received 1, 2, 3, and 4 DEX implants, respectively (Figure 3). The remaining 10 eyes also received ranibizumab (*n* = 49; mean: 4.9; Table 5). In this subgroup, DEX was administered 15 times, and ranibizumab was administered 34 times (Table 5).

On average, 13.35 ± 8.9 days occurred (range 0–30) between diagnosis and the first injection. Mean times between the first and second treatments (T1) and between the second and third treatments (T2) were 5.15 months and 3.7 months, respectively. In the DEX implant cohort, T1 was 5.1 months and T2 was 4.9 months.

### 3.4. Anatomical Response and Therapeutic Decision-Making

At 12 months, macular edema reabsorbed entirely in 12 eyes (32.5%), but persisted in the remaining 25 eyes (67.5%). *I-Macula Web* was instrumental in establishing whether or not a macular edema persisted at each examination and, if present, whether it had increased, decreased, or remained unchanged compared to the previous examination. Data concerning the progression of macular edema were taken into account and related to chosen therapeutic plans. Where the macular edema had decreased, or was absent, a clinician opted against treatment in 92.11% and 97.5% of cases, respectively. Where macular edema had increased, treatment was selected in 66.67% of cases, whereas where the condition had not changed, treatment was repeated in 35.71% of cases only.

## 4. Discussion

This study shows that DEX implant and/or anti-VEGF-based treatment led to statistically significant improvements in visual acuity and CMT in patients with macular edema secondary to RVO. With reference to variation in mean visual acuity, results herein are in keeping with case series reported in the literature. Indeed, Mayer et al. [12] reported on 36 patients treated with DEX implant. After 12 months, mean variations of 6.6 and 7.8 letters were reported in CRVO and BRVO, respectively, though no improvements of over 15 letters are mentioned [12]. Again, whereas our study reports on a cohort of 27 eyes receiving 62 DEX implants overall (mean: 2.9), Mayer et al. reported on a cohort of 38 eyes receiving 80 implants (mean: 2.1) [12]. In our case series, time intervals at T1 and T2 in the subgroup receiving only DEX implant were 5.1 and 4.9 months, respectively, which appear to be in keeping with results by Mayer et al. [12].

Our results confirm that macular edema secondary to RVO is a chronic disease requiring continuing, possibly monthly, monitoring and cyclic injective retreatment. The decision whether to treat or not is basically established by the presence or absence of macular edema. However, it is well known that it is not always possible to reproduce functional and anatomic results by a clinical trial in a real-life setting due to the fact that population in a clinical trial is highly controlled and selected. Interestingly, in 97.5% and 92.1% of our cases, retreatment was ruled out whenever no edema was detected or decreased compared to the previous examination. When macular edema worsened, retreatment was prescribed in 66% of cases; when the disease was stable, retreatment was only prescribed in 35.7% of cases. It is unlikely that the assessment of macular edema was incorrect since clinical evaluations were carried out by trained ophthalmologists with disease-specific experience. For this reason, the fact that only 66% of the time has been given the indication to treatment despite the increased macular edema means that the clinical decision whether to treat or less is often affected by other factors such as patient compliance and waiting list.

The utility of such an electronic clinical platform such as *I-Macula Web* to collect and analyse data proved to be useful for the development of the study and to follow patients in a real-life setting.

## Figures and Tables

**Table 1:** Demography of 35 patients with macular edema secondary to retinal vein occlusion.

Mean age (SD)	72	±10.29
Follow-up (SD)	13.3	±1.3
Gender		
Female	15	42.8%
Male	20	57.2%
Bilateral	2	5.5%
CRVO	17	46.0%
BRVO	20	54.0%

**Table 2:** Distribution by age of 35 patients with macular edema secondary to retinal vein occlusion.

Age (yrs)	Patients	%
40–49	2	5.7
50–59	2	5.7
60–69	9	25.7
70–79	14	40.0

**Table 3:** Number of letters at baseline and 12 months follow-up in 37 eyes with macular edema secondary to retinal vein occlusion treated with DEX implant and/or ranibizumab.

	RVO		CRVO		BRVO	
	Baseline	12 M	Baseline	12 M	Baseline	12 M
Mean	37.9 ± 26	46.9 ± 23	28.2 ± 28	36.5 ± 36	46.1 ± 26	55.7 ± 23
Min–Max	0–80	0–79	3–65	0–74	0–80	0–79
*p*		0.0235		0.1		0.09
Mean change		8.9		8.3		9.5

**Table 4:** Central macular thickness at baseline and 12 months in 37 eyes with macular edema secondary to retinal vein occlusion treated with DEX implant and/or ranibizumab.

	RVO		CRVO		BRVO	
	Baseline	12 M	Baseline	12 M	Baseline	12 M
Mean	436.3 ± 135.5	322.2 ± 71.69	478.9 ± 151.5	342 ± 90.55	400.1 ± 111.5	312.2 ± 57.22
Min–Max	294.6–761.9	226.3–483.1	310.7–761.9	226.3–483.1	294.6–711.6	251.7–461
*p*		<0.0001		<0.0043		<0.0072

**Table 5:** Exposure to drug in 37 eyes with macular edema secondary to retinal vein occlusion treated with DEX implant and/or ranibizumab.

	RVO	CRVO	BRVO
Total (s; m)	37 (111; 2.36)	17 (51; 2.55)	20 (60; 2.2)
DEX (s; m)	27 (62; 2.29)	14 (35; 2.5)	13 (27; 2.07)
DEX + RBZ (s; m)	10 (49; 4.9)	3 (12; 4)	7 (22; 3.14)
DEX	*n* = 15	*n* = 4	*n* = 11
RBZ	*n* = 34	*n* = 12	*n* = 22

s: number of injections; m: mean number of injection.
